# Comparison of single and combined salt and cold stress effects and their challenges for hyperspectral measurements of different *Capsicum* species

**DOI:** 10.1007/s00425-025-04865-0

**Published:** 2025-11-08

**Authors:** Franziska Genzel, Anika Wiese-Klinkenberg

**Affiliations:** 1 Institute of Bio- and Geosciences, Bioinformatics (IBG-4) and Plant Sciences (IBG-2), Forschungszentrum Jülich GmbH, 52425 Jülich, Germany; 2 Bioeconomy Science Center (BioSC), Forschungszentrum Jülich GmbH, 52425 Jülich, Germany

**Keywords:** Abiotic stress, *Capsicum*, Leaf reflectance, Low temperature stress, Salinity, Stress combination

## Abstract

**Main conclusion:**

This study investigates the capability of leaf reflectance measurements to identify stress responses under combined stress treatment.

**Abstract:**

Crops are subjected to various environmental stresses, mostly occurring in combination. Research on combined stresses is important, but most studies focus on single stresses. We analyzed physiological responses of two *Capsicum* species to single cold and salt stresses, which differ from the responses to combinations of these stresses. Combined stress caused growth to decrease more than individual stresses. Single cold stress significantly reduced photosynthetic pigments in both species. However, single salt stress increased pigments in *C. annuum*. Under combined stress, photosynthetic pigments were decreased to a lower extent compared to single cold stress. An increase in leaf reflectance around 550 nm and a significant shift in the red-edge peak of the first derivative corresponded with chlorophyll content. The effects of single cold and combined stress were similar, differing only in magnitude. Only *C. chinense* showed a response in leaf reflectance to salt stress. Spectral vegetation indices distinguished single cold from single salt stress, whereas the effects of single cold and the combined stress were similar, indicating a dominating effect of cold stress. The photochemical reflectance index (PRI), however, distinguished between all three treatments. This research confirms that the responses to combined stresses are unique and different from responses to individual stresses. A strong effect of one stress can mask another. This can lead to misinterpretation when combined stresses occur. The use of hyperspectral signals for quantification of responses to combined stresses must be carefully evaluated and established for further research to assist breeding of climate-resilient crops performing well under multi-stress events.

**Supplementary Information:**

The online version contains supplementary material available at 10.1007/s00425-025-04865-0.

## Introduction

Fruits of *Capsicum* are a rich source of valuable and health-promoting compounds like carotenoids, vitamins, flavonoids, and capsaicinoids (Wahyuni et al. 2013) and their production has been increasing over the last 30 years (FAO 2021). However, suboptimal cultivation conditions impair the production of *Capsicum* fruits. For instance, saline irrigation water and low temperatures are common factors that producers of *Capsicum* have to cope with (Rameshwaran et al. 2015; Ropokis et al. 2019). Species of the genus *Capsicum* show a great variation from highly sensitive to salt tolerant (Aktas et al. 2006). Salt stress symptoms include wilting, senescence, reduced plant growth and fruit yield (Chartzoulakis and Klapaki 2000; Bojórquez-Quintal et al. 2014). *Capsicum* plants are also sensitive to low temperatures resulting in wilting, electrolyte leakage as well as reduced plant growth and fruit yield (Mercado et al. 1997; Korkmaz et al. 2010).

The detection of plant stress during an early phase allows producers to adapt crop management strategies to avoid substantial loss in productivity. In addition, understanding early plant stress responses is highly beneficial for the breeding of stress-tolerant cultivars (Behmann et al. 2014). However, monitoring plant stress in crops is often time-consuming and, depending on the stress factor, symptoms are not always clearly visible, especially during the onset of stress conditions (Lowe et al. 2017). A decrease in chlorophyll content, as it has been observed in chilling-stressed *Capsicum annum* plants within hours after starting the chilling (Guo et al. 2013), would not be visibly detectable at this early time point. Various studies have shown that stress-induced changes in leaf spectral reflectance allow an early detection of plant stress responses (de Jong et al. 2012; Behmann et al. 2014; Lara et al. 2016).

Leaf surface properties, internal cell structure as well as the concentration and distribution of biochemical compounds determine leaf reflectance (Penuelas and Filella 1998). Reflectance changes in the wavelength range from 400 to 700 nm are representative of changes in pigment concentration, mainly chlorophyll and carotenoids. In the near infrared range (700–1300 nm), changes in the internal leaf structure can be detected (Lowe et al. 2017). Abiotic stress leads to changes in leaf structure (Le Gall et al. 2015) and changes in the concentration and composition of foliar pigments (Ramakrishna and Ravishankar 2011; Guo et al. 2013). For instance, a decrease in chlorophyll was observed in *Capsicum* due to salt and cold stress (Guo et al. 2013; Hand et al. 2017). The assessment of chlorophyll content via spectral reflectance measurements enables a rapid estimation of the physiological status of a plant since it is also linked to nitrogen content and photosynthesis (Penuelas and Filella 1998). The rapid, non-destructive detection of differences in the physiological response to stress is also highly beneficial within breeding studies (Behmann et al. 2014). To protect themselves from unfavorable environmental conditions, plants have evolved numerous morphological and physiological stress responses including the accumulation of secondary metabolites (Ramakrishna and Ravishankar 2011; Serrano et al. 2017). Flavonoids, secondary metabolites which also tend to increase due to cold and salt stress in *Capsicum* plants (Hand et al. 2017; Genzel et al. 2021), may confer higher tolerance against abiotic stress conditions (Sharma et al. 2019). So far, research regarding the non-invasive estimation of foliar flavonoids is not as extensive as for chlorophylls and carotenoids; however, a few studies have been conducted, e.g. for chicory (Sytar et al. 2020) and Arabidopsis (Matsuda et al. 2012).

There are several studies investigating the applicability of hyperspectral leaf reflectance measurements for an early detection of stress in *Capsicum* plants exposed to unfavorable abiotic (Wang et al. 2017; Camoglu et al. 2018) or biotic (Herrmann et al. 2017) conditions. In the present study, hyperspectral reflectance measurements were conducted to analyze early responses to cold and salt stress and the combination thereof in two *Capsicum* species, which differ regarding their stress tolerance levels (Genzel et al. 2021). In addition to an early detection of stress responses, the present study aimed at investigating whether differences in stress tolerance between the two species are rapidly detectable using leaf spectral reflectance measurements. A rapid, non-destructive estimation of stress tolerance is highly relevant within breeding trials. Many studies on leaf spectral reflectance focus on the detection of single stresses. The investigation of combinations of stresses is highly relevant as well, since stresses usually occur combined under natural environmental conditions (Choudhury et al. 2017). Here, various effects have been observed from reduction of stress impact in combined stress conditions (Rivero et al. 2014) to intensified additive stress impact (Suzuki et al. 2016) defined as positive or negative interaction, respectively (Mittler 2006). This study aims at investigating how single cold and single salt affect the plant, and whether the combination of both stresses leads to an additive stress impact or if one stress diminishes the impact of the other.

Apart from a complex interaction of stresses in their physiological output, stress combinations might as well cause contrasting impacts on (hyperspectral) indices leading to a misinterpretation or weakening of the signals. A reduction in accuracy for predicting stress has been described when the number of simultaneously applied stresses increased from one single stress to triple stress events (Cotrozzi and Couture 2020). Also, during combined stresses a differentiation and specific quantification of the stress impact of the single factors could be helpful (Ma et al. 2018). Therefore, effects of single as well as combined stress treatment on leaf reflectance spectra were investigated in this study to judge the impact of stress combination on the accuracy of stress quantification using specific hyperspectral patterns and indices. Based on leaf reflectance data, numerous spectral vegetation indices have been developed and used to monitor the performance of plants exposed to abiotic (Carter 1994; de Jong et al. 2012) or biotic (Herrmann et al. 2017; Junker et al. 2019) stress. The objective of this study was to investigate the suitability of leaf spectral reflectance data and of known spectral reflectance indices for the identification of single and combined effects of stress in *Capsicum* plants before the onset of visible symptoms. Furthermore, it was investigated whether these indices allow a separation between genotypes of *Capsicum* that show different levels of stress tolerance.

## Material and methods

### Plant material and stress treatment

Two *Capsicum* species (bell pepper *Capsicum annuum* cv. Mazurka; Rijk Zwaan, De Lier, The Netherlands; chilli *Capsicum chinense* Jacq. CAP1035; Leibniz Institute of Plant Genetics and Crop Plant Research (IPK), Gatersleben, Germany) were sown and cultivated as described (Genzel et al. 2021). Two-month-old *Capsicum* plants were exposed to cold and salt stress and combinations thereof for 14 days (Table 1). The experimental set-up was completely randomized, containing four replicates per treatment (control, cold, 200 mM salt, cold combined with 200 mM salt), species (*C. chinense*, *C. annuum*), and harvest day (1, 7 and 14 days after the start of treatment). For the cold treatment, plants were transferred to an identical growth chamber where the temperature was set at 18 °C/12 °C day/night. For the salt treatment, 200 mM of sodium chloride was added to the Hoagland solution in both growth chambers resulting also in a combined stress treatment of cold and salt (Table 1). Detailed information on the management of temperature and light regime is listed in Table S1.

**Table 1:** Cultivation temperature and salt concentration of the individual stress treatments during 14 days of stress

Condition	Temperature (°C day/night temperature)	Nutrient solution
Control	24/18	Full strength Hoagland solution
Cold	18/12	Full strength Hoagland solution
Salt	24/18	Full strength Hoagland solution containing 200 mM NaCl
Cold plus salt	18/12	Full strength Hoagland solution containing 200 mM NaCl

### Assessment of plant stress—relative growth rate and color

Image-based phenotyping was used to determine stress-induced changes in plant growth and color as described previously (Genzel et al. 2021). Briefly, plants were imaged twice a week starting one day before the beginning of the stress treatment. Projected leaf area (PLA) and red, green, blue (RGB) values were calculated from images of whole plants after processing using a SVM-based image segmentation approach (Briese et al. 2013; Sundgren et al. 2018). The relative growth rate (RGR_PLA_) was calculated according to the following formula:

1 $${\text{RGR}}_{{{\text{PLA}}}} \left( \% \right){ = }\left( {\frac{{{\text{ln PLA2}} - {\text{ln PLA1}}}}{t}} \right) \times {100}{\text{.}}$$

Plant color was determined using RGB values obtained from images for calculating the Excess Greenness Index (ExG_plant_) (Sonnentag et al. 2012):

2 $${\text{ExG}}_{{{\text{plant}}}} = 2G{-}\left( {R + B} \right).$$

It has been shown that abiotic stress causes alterations in leaf structure (Lacerda et al. 2006; Strigens et al. 2013), which may have an effect on reflectance-based estimation of pigments like chlorophyll (Serrano 2008). To assess whether the applied cold and salt treatments affected leaf thickness, we calculated the specific leaf area (SLA) (Strigens et al. 2013),

3 $${\text{SLA }}\left( {{\text{cm}}^{2} /{\text{mg}}} \right) = {\text{LA}}/{\text{LDW}},$$

where LA denotes leaf area (cm^2^, average of the leaf beneath and above the sampling leaf) assessed with the LI-3100C Area Meter (LI-COR Environmental GmbH, Homburg, Germany) and LDW the leaf dry weight (mg, average of the leaf beneath and above the sampling leaf) at harvest. The leaf dry weight, which was also used for the calculation of foliar pigments (Genzel et al. 2021), was determined after drying the leaves for 7 days at 60 °C.

### Destructive determination of leaf pigments

After 1, 7, and 14 days of treatment plants were harvested. Fresh weight was recorded and the leaf beneath the first branching was immediately frozen in liquid nitrogen and stored at − 80 °C. Ground leaf material (Genzel et al. 2021) was used for the extraction of chlorophyll and carotenoids as well as total phenolics and flavonoids.

### Extraction and quantification of chlorophylls and carotenoids

Extraction of chlorophylls and carotenoids was conducted according to the modified protocol described in Junker and Ensminger (2016) by immersing 15–20 mg of ground, frozen leaf material in 2 mL of 98% methanol (100%; VWR International, Langenfeld, Germany) and 2% water buffered with 0.5 M ammonium acetate (Fluka Honeywell, Buchs, Switzerland). After vortex-mixing and shaking for 2 h at 900 rpm at 4 °C, extracts were centrifuged (5 min, 4 °C, at 20,800*g*). The supernatant was diluted with the extraction solution (1:2) and absorbance was measured at 470, 652.4, and 665.2 nm (UV/vis SPECORD 200 PLUS, Analytik Jena, Jena, Germany) which represent the wavelengths of the absorbance maxima of carotenoids and chlorophylls in methanol, respectively. The concentrations of chlorophylls and carotenoids were calculated according to the following Eqs. (4–7) suggested in (Lichtenthaler 1987),

4 $$C_{a} \; \left( {\upmu {\text{g}}/{\text{mL}}} \right) = 16.72A_{665.2} {-}9.16A_{652.4} ,$$

5 $$C_{b} \; \left( {\upmu {\text{g}}/{\text{mL}}} \right) = 34.09A_{652.4} {-}15.28A_{665.2} ,$$

6 $$C_{a + b} \; \left( {\upmu {\text{g}}/{\text{mL}}} \right) = 1.44 \, A_{665.2} + 24.93A_{652.4} ,$$

7 $$C_{x + c}\; \left( {\upmu {\text{g}}/{\text{mL}}} \right) = \left( {1000A_{470} {-}1.63\left[ {C_{a} } \right]{-}104.96\left[ {C_{b} } \right]} \right)/221,$$

where *C*_*a*_, *C*_*b*_, *C*_*a*+*b*_, *C*_*x*+*c*_ denote the concentrations of chlorophyll *a*, chlorophyll *b*, total chlorophyll, and carotenoids and *A*_665.2_, *A*_652.4_, *A*_470_ represent the maximum absorbances at 665.2, 652.4, and 470 nm, respectively.

### Extraction and quantification of phenolic compounds

Phenolic compounds were extracted from 20 mg of ground, frozen leaf material with 2 mL of cold 40:60 (v/v) methanol/water, mixed for 30 min at 900 rpm at 4 °C and centrifuged (20,800*g*, 5 min, 4 °C) as previously described (Genzel et al. 2021). The supernatant was used for quantification of total phenolic and flavonoid content. For determination of the total phenolic (TP) content, an aliquot of the sample supernatant (100 µL) was mixed with 200 µL of 10% Folin–Ciocalteu reagent (Sigma-Aldrich, Darmstadt, Germany) and 800 μL of 700 mM sodium carbonate according to the Folin–Ciocalteu method (Ainsworth and Gillespie 2007; Genzel et al. 2021). After incubation (2 h, room temperature, dark) and subsequent centrifugation (20,800*g*, 1 min, room temperature), the absorbance of the supernatant was measured at 765 nm using the UV/vis SPECORD 200 PLUS. A gallic acid (Sigma-Aldrich) calibration curve was used for quantification of the total phenolic content (expressed in gallic acid equivalents in mg g^−1^ dry weight). For the quantification of flavonoids, an aliquot of the sample supernatant (75 μL) was mixed with distilled water (560 μL), 300 μL of 40:60 (v/v) methanol/water and 40 μL of 5% aluminum chloride (Sigma-Aldrich) and incubated (30 min, room temperature, in the dark) as described (León-Chan et al. 2017; Genzel et al. 2021). The blank contained distilled water instead of aluminum chloride. The absorbance was measured at 405 nm using the UV/vis SPECORD 200 PLUS and flavonoid (TF) content was quantified based on a luteolin (Sigma-Aldrich) calibration curve and was expressed as luteolin equivalents in mg g^−1^ dry weight.

### Spectral measurements

Spectral reflectance of the leaf beneath the first branching was measured with the handheld leaf-clip spectroradiometer PolyPen RP 400 (Photon Systems Instruments, Drasov, Czech Republic) with three technical replicates per leaf (Fig. S1) on each measuring day (1, 4, 7, 11, and 14 days after the start of the stress treatment). The leaf spectral reflectance in the wavelength range from 325.8 to 789.9 nm was measured in comparison to the Spectralon^®^ Reflectance standard (Labsphere Inc., North Sutton, NH, USA). Spectral data were processed in two following steps. First, reflectance data were smoothed using a three-point weighted mean (Danson et al. 1992; Köksal 2011) according to the Eq. (8),

8 $$R_{\lambda i} = 0.5\left( {{\text{Ref}}_{\lambda i} } \right) + 0.25\left( {{\text{Ref}}_{\lambda i} + 1} \right) + 0.25({\text{Ref}}_{\lambda i} - 1),$$

where *R*_*λi*_ denotes smoothed reflectance at wavelength *λi* (nm). Subsequently, the first derivative of smoothed reflectance data was calculated using the following Eq. (9) according to (Shibayama et al. 1993; Köksal 2011)

9 $$\rho_{\lambda i} = \frac{{\left( {\mathop \sum \nolimits_{j = i + 1}^{j = i + 3} R_{\lambda i} - \mathop \sum \nolimits_{j = i - 1}^{j = i - 3} R_{\lambda i} } \right)/3}}{\Delta \lambda },$$

where *ρ*_*λi*_ is the first derivative at wavelength *λi* (nm) with an interval of two consecutive bands (Δ*λ*) of 1.8 nm.

A selection of spectral vegetation indices was assessed based on literature research as shown in Table 2 (Gitelson and Merzlyak 1997; Gamon and Surfus 1999; Barnes et al. 2000; Zarco-Tejada et al. 2001; Poss et al. 2006; de Jong et al. 2012; Quemada et al. 2014).

**Table 2:** Selected spectral reflectance indices investigated in this study

Spectral reflectance index	References
Red/Green Index (RGI)	*R*_690_/*R*_550_ (de Jong et al. 2012)
Photochemical Reflectance Index (PRI)	(*R*_531_ − *R*_570_)/(*R*_531_ + *R*_570_) (Gamon and Surfus 1999)
Normalized Difference Red Edge Index (NDRE)	(*R*_790_ − *R*_720_)/(*R*_790_ + *R*_720_) (Barnes et al. 2000)
Normalized Difference Vegetation Index (NDVI)	(*R*_750_ − *R*_705_)/(*R*_750_ + *R*_705_) (Gamon and Surfus 1999)
Green Region Normalized Difference Vegetation Index (NDVI_green_)	(*R*_550_ − *R*_670_)/(*R*_550_ + *R*_670_) (Poss et al. 2006)
Zarco-Tejada and Miller Index (ZMI)	*R*_750_/*R*_710_ (Zarco-Tejada et al. 2001)
Gitelson and Merzlyak Index (GM1)	*R*_750_/*R*_550_ (Gitelson and Merzlyak 1997)
FLAV_700,760_	(0.790 × *R*_700_)/(*R*_760_ + 0.40) (Quemada et al. 2014)

### Statistical analyses

Statistical analyses were performed using SigmaStat in Sigma Plot 13 (Systat Software, San Jose, CA, USA). Depending on the results of tests for homogeneity of variance (Brown–Forsythe test) and normal distribution (Shapiro–Wilk test), the data were examined using one-way analysis of variance (ANOVA) or Kruskal–Wallis one-way ANOVA on ranks, followed by Tukey’s HSD post hoc test or Dunn’s test for the comparison of means, respectively. Correlations between values of plant traits (RGR_PLA_, ExG_plant_), foliar pigment concentrations, and spectral data of individual plants were assessed using Pearson’s correlation coefficients.

## Results

### Cold and salt stress differentially affect the growth and greenness of *C. annuum* and *C. chinense*

**Fig. 1:**
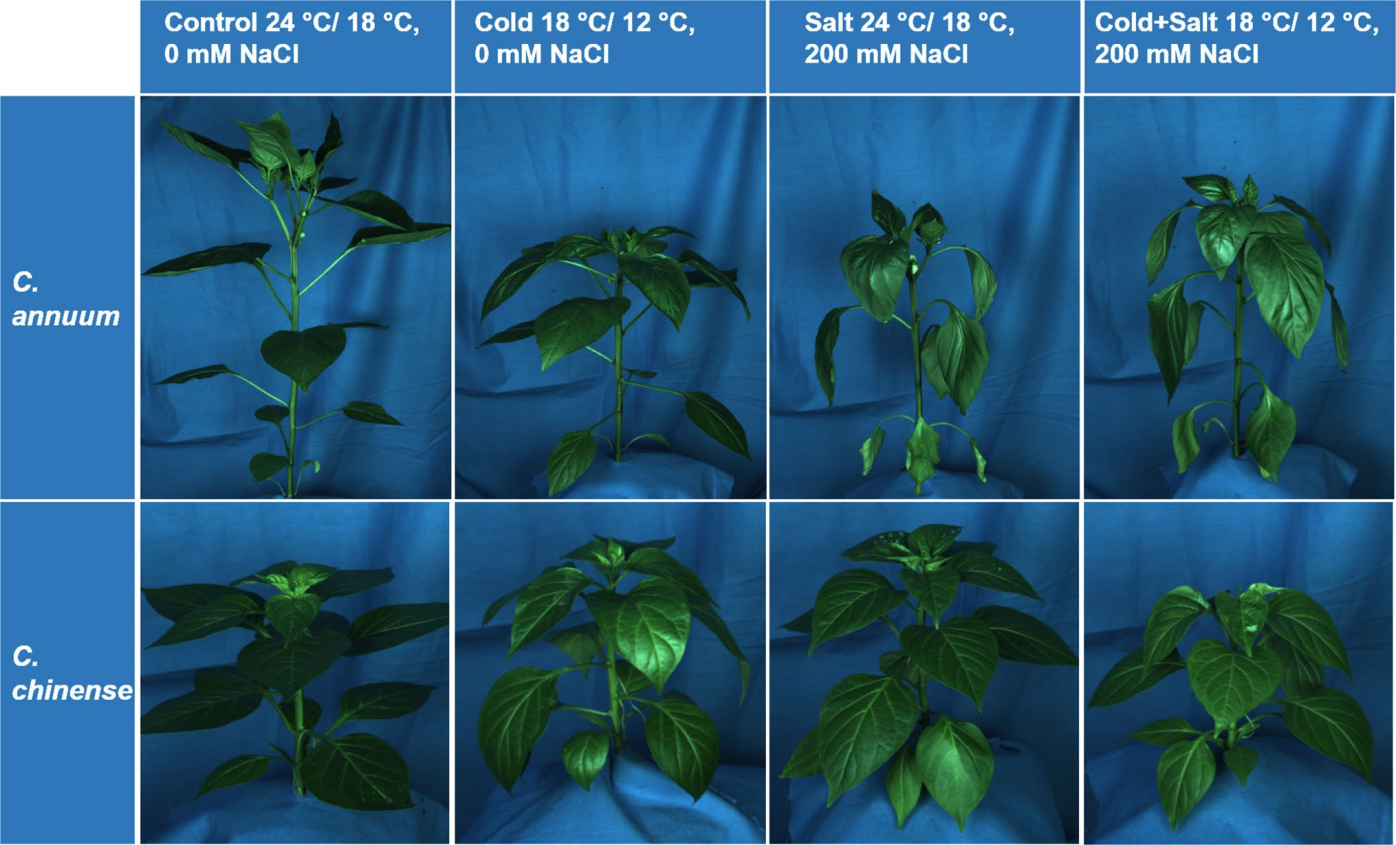
Images show randomly selected plants of *C. annuum* and *C. chinense* of the respective treatments after 13 days of stress treatment. Images were used for the assessment of relative growth rate based on projected leaf area and plant color; for better visibility images have been brightened here

**Fig. 2:**
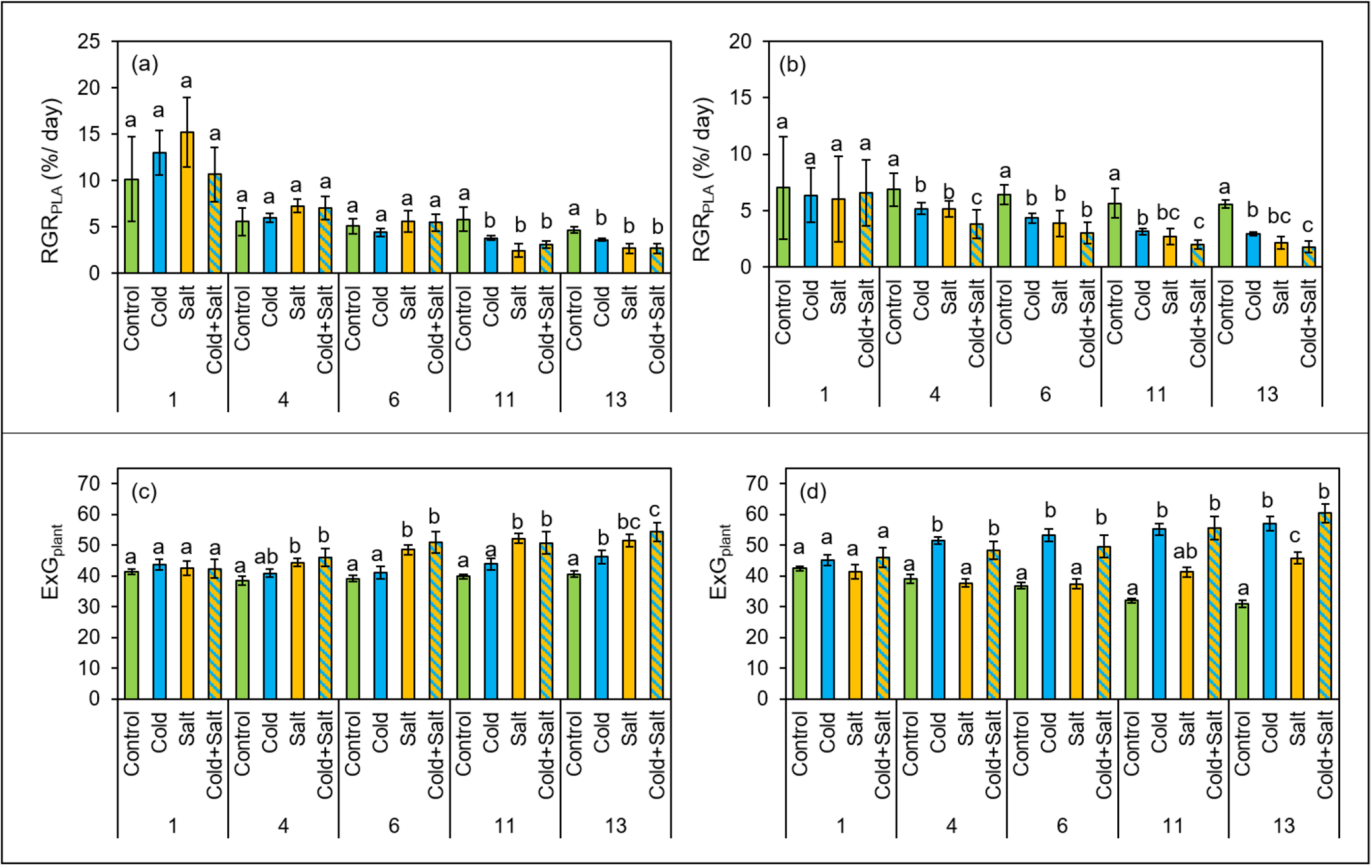
Image-based phenotyping used for assessment of relative growth rate based on projected leaf area (%/day, RGR_PLA_) and plant color (expressed as ExG_plant_) of plants of *C. annuum* (**a**, **c**) and *C. chinense* (**b**, **d**) over the time course of the stress treatment. Data show means with standard deviations (*n* = 4 plants). Different letters indicate significant differences between treatments (control, cold, salt, cold plus salt) per species within each time point (*P* ≤ 0.05)

The impact of the cold and salt treatments on the performance of two *Capsicum* species was assessed throughout the time course of 14 days of stress treatment using image-based phenotyping. Figure 1 reveals a visual wilting response for the *C. annuum* plants due to single salt stress and the combination of cold and salt, but not due to single cold. In contrast, the *C. chinense* plants showed only a slight wilting in response to all three treatments. The treatments with cold, salt and their combination significantly reduced the relative growth rate (RGR_PLA_) in both species (Fig. 2a, b). However, the reduction occurred much earlier in the *C. chinense* plants (after 4 days) than in the *C. annuum* plants (after 11 days). In line with this, a significant reduction of plant dry weight (DW) was detected after 14 days (Fig. S2). Furthermore, RGR_PLA_ was significantly lower for the combination of cold and salt stress compared to single cold stress in *C. chinense* (Fig. 2b).

Differences in plant color were not clearly visible after 14 days of stress treatment. However, the color index excess greenness, which was calculated from Red Green Blue values (RGB) extracted from the images of plants (ExG_plant_), revealed some significant differences in the greenness of the plants between stressed and unstressed plants for both species already after 4 days (Fig. 2). Plants of *C. annuum* showed an increased ExG_plant_ after single salt treatment and after a combination of cold and salt treatment compared to control, and later also in comparison to the almost unchanged ExG_plant_ of cold-treated plants (Fig. 2c). For *C. chinense,* single cold application and also its combination with salt caused a significant increase in the ExG_plant_ compared to control and salt plants (Fig. 2d).

In contrast to *C. annuum*, which showed an early significantly increased ExG_plant_ after single salt treatment already after 4 days (Fig. 2c), *C. chinense* responded rather late to single salt stress with a significantly increased ExG_plant_ after 13 days (Fig. 2d). Similarly, *C. annuum* increased the ExG_plant_ in response to cold not before 13 days (Fig. 2c). After 13 days, the ExG_plant_ was significantly increased in both species for all treatments (Fig. 2). At this time point, ExG_plant_ was highest for single salt treatment and the combination with cold in *C. annuum*, while in *C. chinense* single cold and the combination with salt caused the highest ExG_plant_ (Fig. 2).

### Stress-induced changes of concentrations of foliar pigments

Single cold treatment significantly reduced total chlorophyll and carotenoid content in both species compared to plants grown under control conditions after 7 and 14 days (Tables 3, 4). In contrast, single salt treatment caused a significantly higher content of total chlorophylls and carotenoids in *C. annuum* compared to the control after 7 days and increased the content in tendency after 14 days (Table 3). Single salt treatment did not affect the content of total chlorophylls or carotenoids in the *C. chinense* plants (Table 4). The combination of the two stresses decreased chlorophylls and carotenoids in both species in tendency, but far less than the treatment with cold alone, and only significantly after longer treatment (14 days) in *C. chinense* for chlorophyll.

**Table 3:** Foliar concentration of total chlorophylls (*a* + *b*), carotenoids, total phenolics, flavonoids, and specific leaf area of plants of *C. annuum* grown under different conditions (control, cold, salt, cold plus salt) after 1, 7, and 14 days

Parameter	Day	Control	Cold	Salt	Cold + salt
*C. annuum*					
Chlorophyll *a* + *b* (mg/g DW)	0	19.91 ± 4.6			
	1	16.22 ± 0.96 ab	14.26 ± 0.92 b	19.27 ± 2.14 a	15.90 ± 2.10 ab
	7	14.60 ± 0.35 a	**8.91 ± 0.78 b**	**18.48 ± 2.24 c**	12.82 ± 0.90 a
	14	13.38 ± 0.96 ac	**7.54 ± 0.41 b**	16.62 ± 1.81 a	11.28 ± 2.59 bc
Carotenoids (mg/g DW)	0	5.80 ± 1.57			
	1	4.70 ± 0.29 ab	4.27 ± 0.33 b	5.49 ± 0.56 a	4.71 ± 0.55 ab
	7	4.45 ± 0.13 a	**2.85 ± 0.20 b**	**5.50 ± 0.54 c**	3.96 ± 0.26 a
	14	3.87 ± 0.30 ac	**2.38 ± 0.21 b**	4.78 ± 0.41 a	3.42 ± 0.88 bc
Chlorophyll *a*/*b* ratio	0	6.65 ± 0.83			
	1	6.42 ± 0.42	7.19 ± 1.21	6.74 ± 1.15	6.69 ± 0.54
	7	7.87 ± 0.22	8.36 ± 1.73	7.35 ± 0.62	8.08 ± 0.83
	14	6.10 ± 0.1 a	**7.91 ± 0.7 b**	6.77 ± 0.6 ab	7.28 ± 0.5 ab
Carotenoids/chlorophyll ratio	0	0.289 ± 0.012			
	1	0.290 ± 0.006	0.299 ± 0.011	0.285 ± 0.007	0.297 ± 0.007
	7	0.305 ± 0.003	0.321 ± 0.012	0.299 ± 0.007	0.309 ± 0.007
	14	0.289 ± 0.003 a	**0.315 ± 0.014 b**	0.288 ± 0.007 a	0.301 ± 0.013 ab
Total phenolics (mg/g DW)	0	15.09 ± 2.54			
	1	13.44 ± 1.94	17.12 ± 2.96	17.07 ± 1.89	16.83 ± 1.22
	7	11.28 ± 1.46	12.16 ± 4.02	17.08 ± 2.58	19.19 ± 0.89
	14	8.04 ± 1.32	13.86 ± 3.68	13.20 ± 4.59	16.24 ± 2.41
Total flavonoids (mg/g DW)	0	12.50 ± 1.98			
	1	6.04 ± 1.25	7.59 ± 0.10	7.95 ± 1.62	7.59 ± 0.10
	7	5.27 ± 0.45 a	6.85 ± 2.44 ab	9.09 ± 1.74 ab	**12.13 ± 0.83 b**
	14	3.76 ± 0.74 a	**8.76 ± 2.28 b**	5.54 ± 2.47 ab	**9.07 ± 1.38 b**
Specific leaf area (cm^2^/mg)	0	0.35 ± 0.03			
	1	0.41 ± 0.17	0.25 ± 0.02	0.34 ± 0.05	0.32 ± 0.02
	7	0.23 ± 0.05	0.19 ± 0.02	0.25 ± 0.03	0.20 ± 0.02
	14	0.22 ± 0.00	0.16 ± 0.01	0.22 ± 0.01	0.20 ± 0.03

Data are means ± standard deviations (*n* = 4 plants). Significant differences between treatments within each time point are indicated by different letters (*P* ≤ 0.05). Values, which are different from control are shown in bold

**Table 4:** Foliar concentration of total chlorophylls (*a* + *b*), carotenoids, total phenolics, flavonoids, and specific leaf area of plants of *C. chinense* grown under different conditions (control, cold, salt, cold plus salt) after 1, 7, and 14 days

Parameter	Day	Control	Cold	Salt	Cold + salt
*C. chinense*					
Chlorophyll *a* + *b* (mg/g DW)	0	11.01 ± 0.71			
	1	10.59 ± 0.98	9.78 ± 1.12	10.68 ± 1.39	12.86 ± 6.03
	7	10.93 ± 0.96 a	**6.68 ± 1.04 b**	10.55 ± 1.68 a	8.35 ± 0.80 ab
	14	10.94 ± 0.70 a	**5.84 ± 0.46 b**	9.61 ± 1.76 a	**7.22 ± 0.43 b**
Carotenoids (mg/g DW)	0	3.26 ± 0.12			
	1	3.08 ± 0.32	2.93 ± 0.36	3.07 ± 0.42	3.89 ± 1.81
	7	3.26 ± 0.26 a	**2.16 ± 0.32 b**	3.03 ± 0.47 a	2.60 ± 0.22 ab
	14	3.05 ± 0.26 a	**1.89 ± 0.18 b**	2.75 ± 0.58 a	2.40 ± 0.17 ab
Chlorophyll *a*/*b* ratio	0	8.00 ± 1.46			
	1	8.23 ± 1.29	7.83 ± 0.89	7.66 ± 0.98	8.14 ± 0.19
	7	7.88 ± 0.43	10.06 ± 1.06	8.01 ± 1.02	8.95 ± 1.81
	14	6.67 ± 0.39	8.32 ± 0.50	7.33 ± 0.74	8.23 ± 1.24
Carotenoids/chlorophyll ratio	0	0.296 ± 0.014			
	1	0.290 ± 0.006	0.299 ± 0.006	0.287 ± 0.008	0.303 ± 0.005
	7	0.299 ± 0.006 ab	0.324 ± 0.014 a	**0.287 ± 0.001 b**	0.312 ± 0.009 ab
	14	0.278 ± 0.012 a	0.324 ± 0.011 ab	0.285 ± 0.013 ab	**0.332 ± 0.005 b**
Total phenolics (mg/g DW)	0	10.18 ± 1.12			
	1	12.08 ± 2.12	12.97 ± 0.97	9.81 ± 1.71	12.94 ± 0.96
	7	7.15 ± 1.56	9.26 ± 2.47	13.30 ± 2.82	13.30 ± 1.91
	14	2.76 ± 0.67 a	7.76 ± 2.91 ab	7.29 ± 2.54 ab	**13.70 ± 1.91 b**
Total flavonoids (mg/g DW)	0	5.26 ± 1.37			
	1	4.09 ± 0.87	4.48 ± 0.62	3.52 ± 0.52	3.83 ± 0.49
	7	1.85 ± 0.32 a	4.42 ± 1.30 ab	4.08 ± 1.15 ab	**4.79 ± 1.50 b**
	14	1.16 ± 0.23 a	**3.95 ± 1.31 bc**	1.67 ± 0.37 ab	**5.35 ± 1.83 c**
Specific leaf area (cm^2^/mg)	0	0.35 ± 0.01			
	1	0.34 ± 0.05	0.30 ± 0.01	0.31 ± 0.01	0.27 ± 0.02
	7	0.25 ± 0.01	0.20 ± 0.01	0.28 ± 0.02	0.22 ± 0.04
	14	0.19 ± 0.05	0.18 ± 0.00	0.20 ± 0.06	0.26 ± 0.01

Data are means ± standard deviations (*n* = 4 plants). Significant differences between treatments within each time point are indicated by different letters (*P* ≤ 0.05). Values, which are different from control are shown in bold

Only in *C. annuum,* changes in total chlorophyll content were accompanied by a significant increase in the chlorophyll a to chlorophyll b ratio due to cold treatment after 14 days (Table 3). The carotenoid to chlorophyll ratio was also changed in *C. annuum* due to single cold treatment after 14 days indicating changes in the photosystems and photosynthetic activity. In contrast, in *C. chinense* significant changes in the light harvesting pigment composition were seen for the carotenoid to chlorophyll ratio only in the combined cold and salt treatment after 14 days (Table 4).

The content of total phenolics and flavonoids increased in tendency in both species upon all three stress treatments (Tables 3, 4). However, a significant increase of total phenolics compared to plants grown under control conditions was only observed in the *C. chinense* plants after 14 days of combined stress treatment (Table 4). The effect of stress treatments was more pronounced for the accumulation of flavonoids. Single cold stress increased flavonoids significantly after 14 days in both species (Tables 3, 4). The combination of cold and salt significantly increased the content of flavonoids in *C. chinense* after 7 and 14 days; however, in *C. annuum* flavonoids were only significantly increased after 14 days of combined stress (Tables 3, 4). In contrast, single salt treatment increased flavonoids only in tendency. Furthermore, in Tables 3 and 4 we show the specific leaf area (SLA) of the analyzed leaves, because leaf thickness might affect chlorophyll estimation via leaf reflectance measurements (Serrano 2008). SLA shows tendencies to be reduced due to cold treatment in both species.

### Alterations of leaf reflectance upon stress treatment

**Fig. 3:**
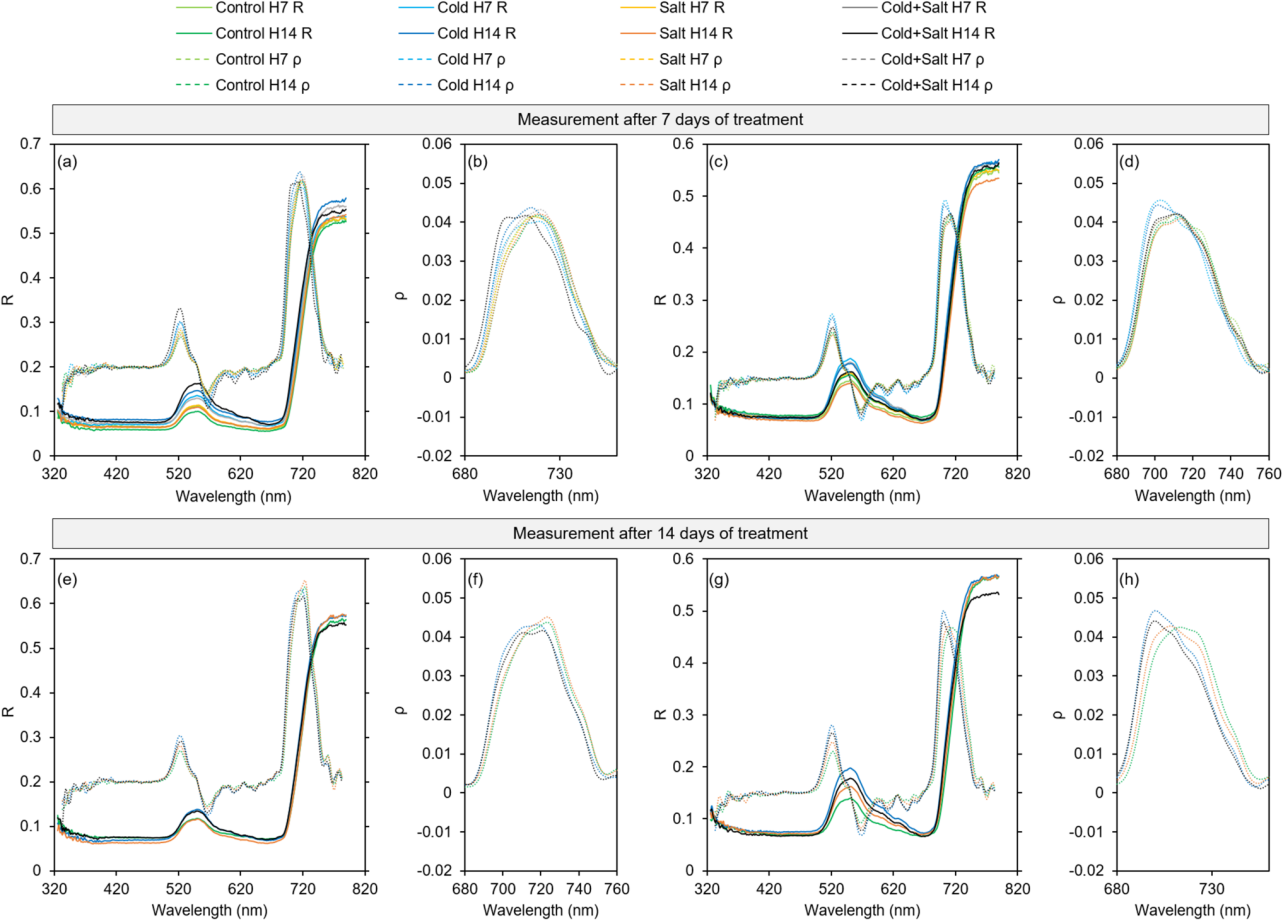
Mean (*n* = 4 plants with 3 averaged technical replicates per plant) spectral reflectance (*R*) and mean first derivative of spectral reflectance (*ρ*) of leaves of *C. annuum* (**a**, **e**) and *C. chinense* (**c**, **g**) after 7 days (**a**, **c**; here two groups per treatment are shown including plants harvested after 7 (H7) and 14 (H14) days for destructive measurements, respectively) and after 14 days of stress treatment (**e**, **g**). Zoom in on the red edge region of the first derivative shown for *C. annuum* after 7 (**b**) and 14 days (**f)** and for *C. chinense* after 7 (**d**) and 14 days (**h**) of stress treatment

Spectral reflectance and first derivative of reflectance of leaves of the two species *C. annuum* and* C. chinense*, which were cultivated under control and stress conditions for 7 and 14 days, are shown in Fig. 3. An increased reflectance in the wavelength range of 530–630 nm due to the treatment with single cold and the combination of cold and salt is detectable for both species already after 7 days (Fig. 3a, c) and more pronounced after 14 days of treatment (Fig. 3e, g). Similarly, the first derivative of spectral reflectance allows a clear separation between control and treatments involving cold stress with the highest difference at 521.7 nm and in the range from 701 to 718.7 nm in both species, especially after 14 days of treatment (Fig. 3e, g). Although to a lower extent, differences in reflectance and in the first derivative of reflectance within this wavelength range were also observed between control and single salt treatment in *C. chinense* (Fig. 3c, g), but not in *C. annuum* (Fig. 3a, e). No distinct differences between the treatments were observed for specific wavelengths. However, differences in the first derivative around 690 to 750 nm, the red edge position, were observed between the treatments in both species (Fig. 3b, d, f, h). After 14 days, there is a displacement of the red edge peak to lower wavelengths for single cold (5.2 nm) and the combined stress treatment (3.5 nm) detectable in *C. annuum* (Fig. 3b, f). This displacement to lower wavelengths is stronger and earlier seen in *C. chinense*, with a shift of 8.8 nm for cold and the combined treatment after 7 days (Fig. 3d) and a shift of 12.4 nm for cold and the combined treatment, respectively, after 14 days (Fig. 3h). In contrast to *C. annuum*, there is also a small shift in the peak to lower wavelengths (5.3 nm) detectable for single salt treatment in *C. chinense* after 14 days (Fig. 3h).

### Changes in spectral reflectance indices upon stress treatment

**Fig. 4:**
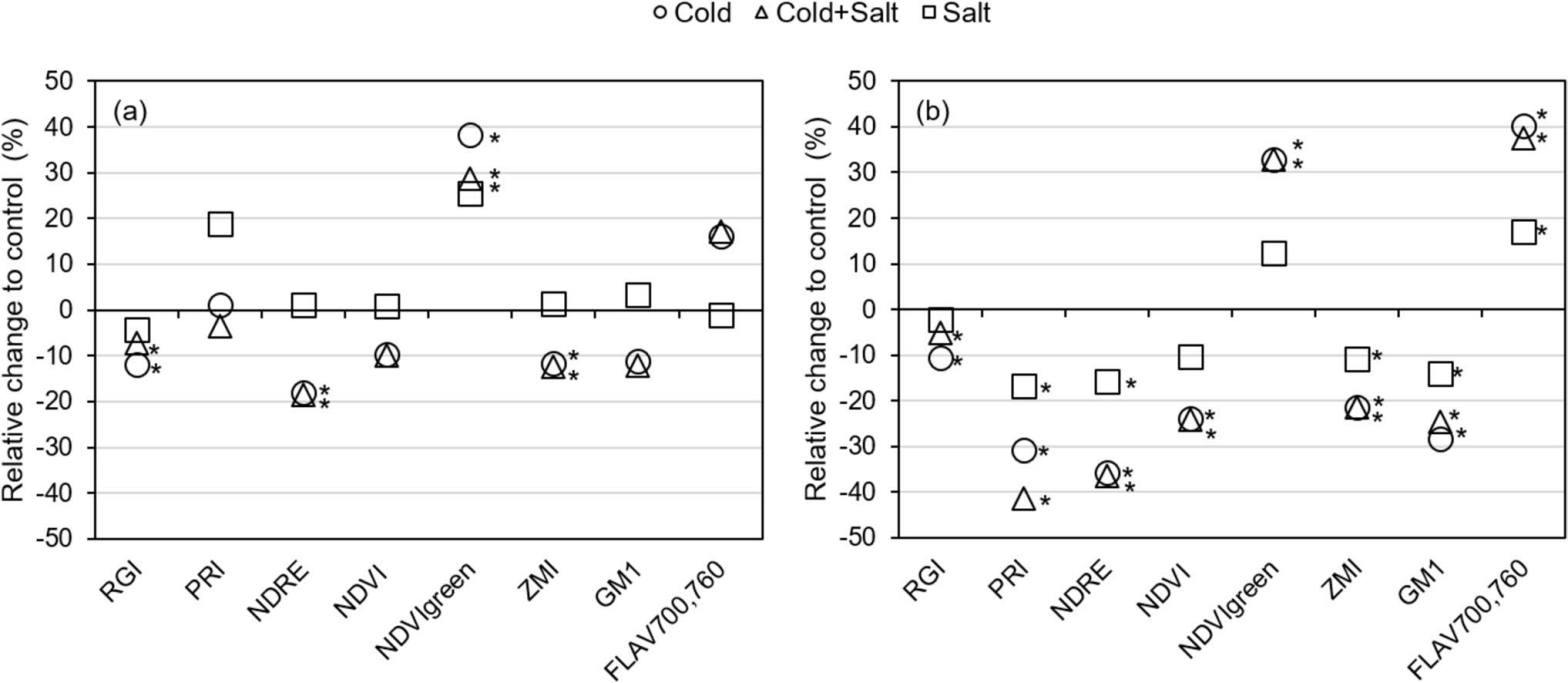
Change of selected hyperspectral indices upon stress treatment (cold, salt, combination of cold and salt) relative to control shown for *C. annuum* (**a**) and *C. chinense* (**b**) after 14 days of stress treatment. Significant differences between control and stress treatments (cold, salt, cold plus salt) are indicated by asterisks (*P* ≤ 0.05)

In general, cold and the combination of cold and salt changed the evaluated spectral reflectance indices by a similar magnitude (Fig. 4).The photochemical reflectance index (PRI) in *C. chinense* was an exception. This index was significantly reduced due to the applied stress treatments with an increasing reduction from salt over cold to the combination of cold and salt (Fig. 4b) showing a clear additive effect of the single treatments. In *C. annuum*, single cold stress and its combination with salt had a much stronger effect on the analyzed indices than single salt treatment, except for PRI. Moreover, PRI rather specifically increased only in response to salt stress in *C.* *annuum;* however, not significantly (Fig. 4a). In *C. annuum*, a significant effect of single salt treatment was only observed for NDVI_green_ and the Zarco-Tejada and Miller index (ZMI) (Fig. 4a), whereas in *C. chinense* PRI, the normalized difference red edge index (NDRE), ZMI, Gitelson and Merzlyak index 1 (GM1), and Flav_700,760_ were significantly changed by single salt stress (Fig. 4b). Values of NDVI_green_, and NDRE were strongly affected by stress treatments compared to controls in both species with *C. chinense* showing generally a more pronounced effect on indices than *C. annuum* (Fig. 4). For instance, NDRE decreased significantly in *C. annuum* due to cold and the combination with salt (approximately 18%) and in *C. chinense* due to all three treatments (cold: 35.7%, salt: 15.9%, combination: 36.5%). All the analyzed indices were significantly affected by single cold treatment and the combination of cold and salt in *C. chinense*, including Flav_700,760_ with the strongest reaction, with changes of ca. 35–40% relative to the control (Fig. 4b). In contrast, only the red-green index (RGI), NDRE, NDVI_green_, and ZMI were significantly changed upon cold and its combination with salt in *C. annuum* (Fig. 4a).

### Relationships between spectral reflectance features, plant morphology, and foliar pigments

**Fig. 5:**
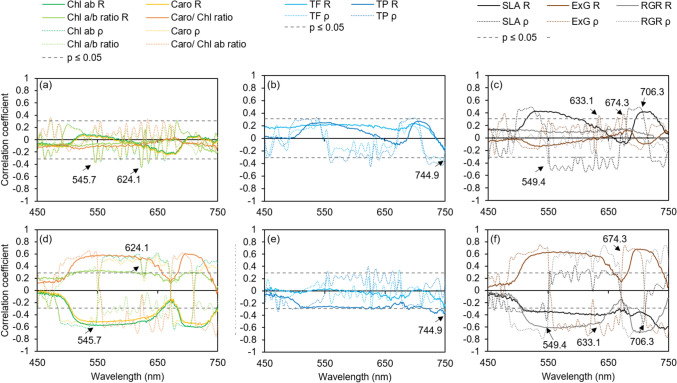
Correlation analysis between the spectral reflectance (solid line, *R*), first derivative of spectral reflectance (dotted line, *ρ*) and content of chlorophylls (chl *ab*), carotenoids (caro), chlorophyll *a* to *b* ratio (chl *a*/*b* ratio), carotenoid to chlorophyll ratio (caro/chl ratio) (**a**, **d**), total flavonoids (TF) and total phenolics (TP) (**b**, **e**), SLA, ExG_plant_, and RGR_PLA_ (**c**, **f**) in *C. annuum* (**a**–**c**) and *C. chinense* (**d**–**f**). Dashed line indicates *P* ≤ 0.05

To use hyperspectral data for quantification of stress reactions, we analyzed the correlation of the hyperspectral data to the quantified stress responses of plant growth (RGR_PLA_), morphology (SLA) and color (ExG_plant_) as well as to the content of foliar pigments. Coefficients of correlation obtained for the first derivative reached higher values than for original reflectance data (Fig. 5). Only SLA showed a significant (*P* ≤ 0.05) positive (*C. annuum*) and negative correlation (*C. chinense*) with leaf reflectance values of broad wavelengths simultaneously in both species (Fig. 5c and f). ExG_plant_ and RGR_PLA_ correlated well with a broad range of the wavelengths in *C. chinense.*

Significant correlations (*P* ≤ 0.05) for values of the first derivative occurred in both species at the same wavelengths and were detected for several quantified stress response indicators (Fig. 5). Although there was some overlapping regarding significant correlation between specific wavelengths and individual plant traits, larger differences were observed between the species.

First, *C. chinense* showed good correlations between the first derivative of reflectance and e.g. chlorophylls (chl_*ab*_, *R* = − 0.65 at 677.9 nm), caro/chl_*ab*_ ratio (*R* = − 0.75, 746.7 nm), ExG_plant_ (*R* = − 0.78, 746.7 nm) and RGR_PLA_ (*R* = − 0.80, 540.2 nm), whereas correlations in *C. annuum* were rather low for all measured plant traits. Secondly, the species showed divergent correlations. For instance, in *C. annuum* SLA and reflectance in the range of 514–598 nm correlated positively, whereas in *C. chinense* a negative correlation was observed within this region (Fig. 5). In both species, significant correlations (*P* ≤ 0.05) were detected in the regions 495–590 nm, 620–680 nm, and around 740–750 nm (Fig. 5). More specifically, significant correlations with the first derivative of reflectance were detected for the chlorophylls at 545.7 and 624.1 nm, for the TF at 744.9 nm, for RGR_PLA_ at 549.4 nm, for ExG_plant_ at 633.1 and 674.3 nm and for SLA at 706.3 nm in both species (indicated by arrows in Fig. 5).

**Fig. 6:**
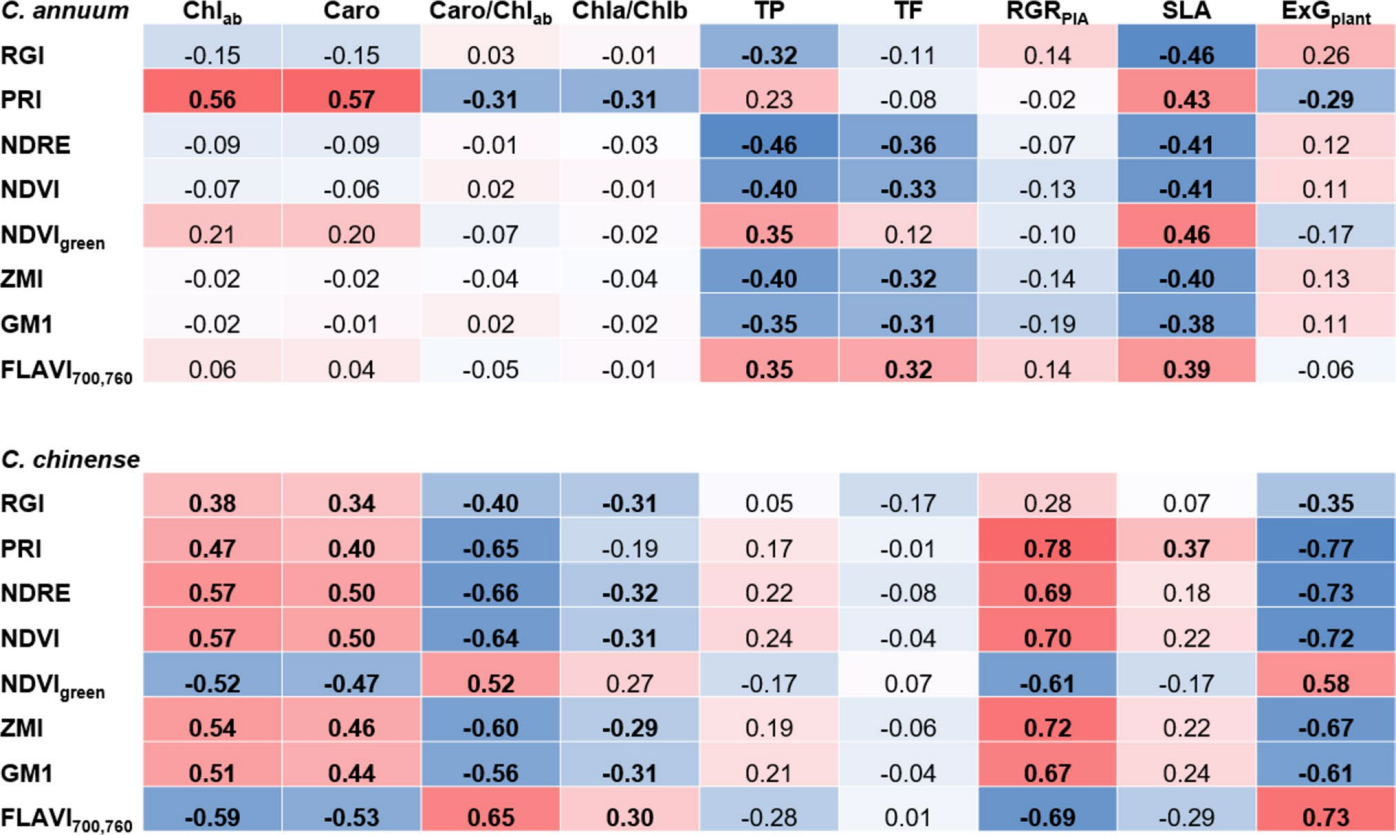
Coefficient (*R*, Pearson) of determination of relationships between reflectance indices and stress indicators [total chlorophylls (Chl_*ab*_, mg/g DW), carotenoids (Caro, mg/g DW), the ratio of carotenoids to chlorophylls (Caro/Chl_*ab*_ ratio), the ratio of chlorophyll *a* to *b* (Chl *a*/*b* ratio), total phenolics (TP, mg/g DW), total flavonoids (TF, mg/g DW), relative growth rate (RGR_PLA_, %/day), specific leaf area (SLA, cm^2^/mg), and Excess Greenness Index (ExG_plant_)] of *C. annuum* and *C. chinense* including data from 1, 7, and 14 days after the start of stress treatment. Significance (*P* ≤ 0.05) is indicated in bold

In Fig. 6 the Pearson’s correlation coefficient (*R*) shows the relationship between known reflectance indices and quantified stress response indicators for *C. annuum* and *C. chinense*, respectively. For *C. annuum*, correlations between reflectance indices and the plant parameters TF, TP, and SLA were significant for most of the indices and also stronger compared to *C. chinense* (Fig. 6). In contrast, the stress indicators chlorophyll and carotenoid content, the chl *a*/*b* ratio, the caro/chl ratio, ExG_plant_, and RGR_PLA_ showed significant and often higher correlations with reflectance indices in *C. chinense* than in *C. annuum*. The SLA correlated significantly (*P* ≤ 0.05) with all tested indices in *C. annuum* (Fig. 6), whereas in *C. chinense* a significant correlation (*P* ≤ 0.05) was only observed for PRI. In *C. annuum*, a significant correlation between concentrations of total chlorophyll and carotenoids and reflectance indices was only observed for PRI, whereas in *C. chinense* all tested reflectance indices correlated significantly with these photosynthesis pigments and with the caro/chl_*ab*_ ratio and in most cases also with the chl_*a*_/chl_*b*_ ratio. To further investigate the impact of single stress treatments on the correlation between stress parameters and spectral indices, separate correlation analyses were conducted for the three treatments (control with cold, control with salt, control with a combination of cold and salt, Fig. S3). In *C. annuum*, a significant correlation with chlorophylls was only observed for NDVI_green_ and RGI for the combined stress treatment and for PRI and when single cold or the combined treatment were included, whereas no significant correlation was detected between spectral indices and the caro/chl_*ab*_ ratio or the chl_*a*_/chl_*b*_ ratio (Fig. S3). In *C. chinense,* however, a strong significant correlation with chlorophylls was observed when the single cold treatment was regarded, whereas there was no correlation detectable for the single salt treatment (Fig. S3). When the combined stress treatment was considered, a significant but lower correlation with photosynthetic parameters was detected compared to single cold in *C. chinense*. TP and TF correlated significantly only with spectral indices in *C. annuum*, not in *C. chinense*. The RGR_PLA_ was not significantly correlated with spectral indices in *C. annuum*, while in *C. chinense* a highly significant correlation was detected, which even increased for most of the tested indices when stress treatments were combined (Fig. S3). Again, the correlation for single cold was higher in *C. chinense* compared to single salt. When considering the combined stress treatment, the SLA correlated significantly with all studied spectral indices in *C. annuum*, whereas no significant correlation was observed in *C. chinense*. The correlation between ExG_plant_ and spectral indices was only significant for PRI for the combined treatment in *C. annuum*, whereas a significant correlation was detected in *C. chinense* for single cold and the combined stress treatment for most of the indices evaluated (Fig. S3).

**Fig. 7:**
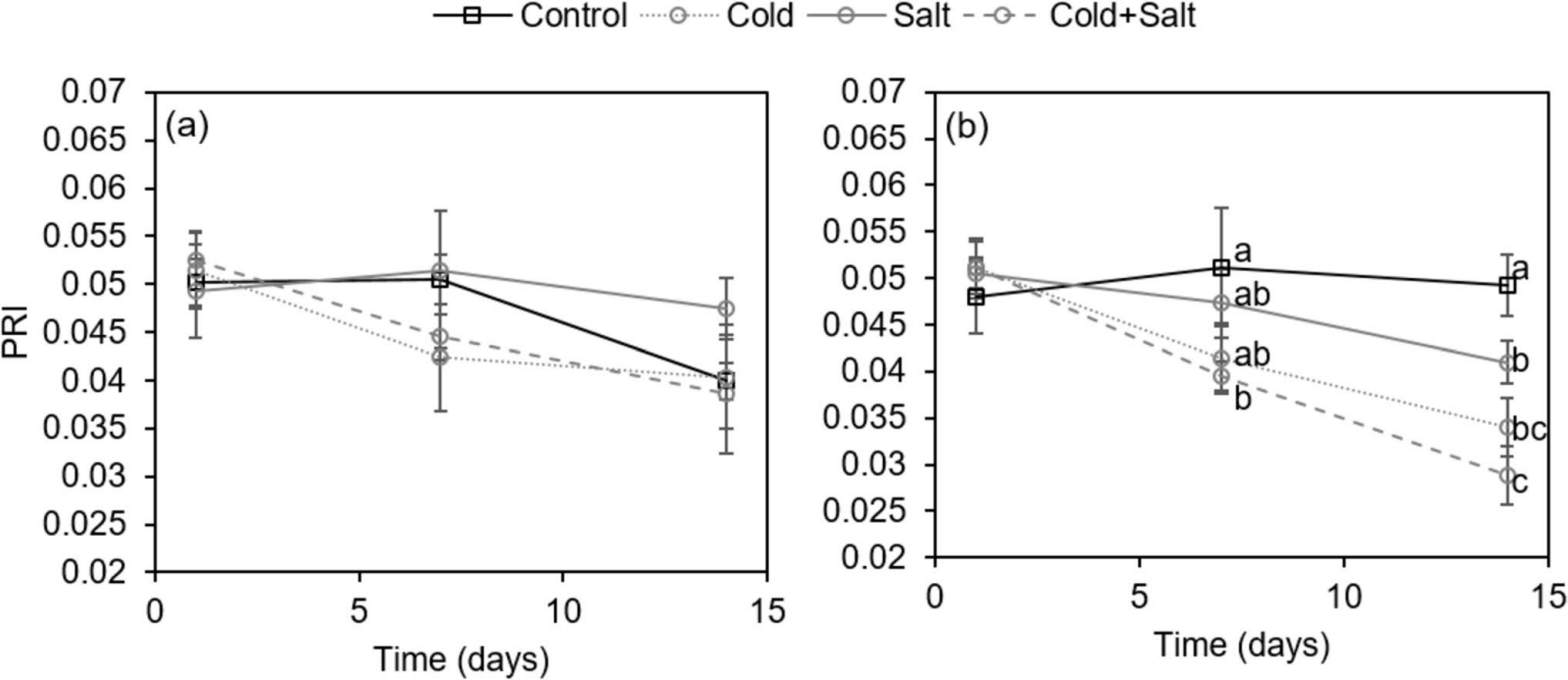
Temporal changes of photochemical reflectance index (PRI) of *C. annuum* (**a**) and *C. chinense* (**b**) leaves grown under control conditions and after treatment with cold, salt, and cold plus salt (cold + salt) after 1, 7, and 14 days. Data are means ± standard deviations (*n* = 4 plants). Letters a, b, c indicate significant differences between treatments within each time point (*P* ≤ 0.05)

The index PRI stood out from the other investigated indices, because it significantly correlated with chlorophyll and carotenoid content, the ratio caro/chl_*ab*_, and SLA in both species consistently (Fig. 6) and was therefore analyzed over the time course of the experiment (Fig. 7). Also, the change of PRI for stress treatments relative to control showed the best separation of the three treatments after 14 days (Fig. 4). Both species show distinct differences regarding the temporal development and the response to the respective treatments (Fig. 7). In *C. annuum*, PRI shows a decreasing trend over time in all treatments except for single salt (Fig. 7a). Still, treatments do not differ significantly regarding the PRI in this case (Fig. 7a). In *C. chinense*, PRI of leaves of control plants does not change over the time course of the experiment, whereas PRI of leaves of all stress treatments decreases over time leading to a significantly lower PRI after 14 days compared to the control (Fig. 7b). Thus, an early significant effect for the combined stress was seen after 7 days and a distinct separation between the combined treatment effect and the salt stress effect was possible after 14 days (Fig. 7b).

## Discussion

In this study, we compared the effect of cold, salt, and combined stress treatments on the spectral reflectance of leaves of two *Capsicum* species over a time course of 14 days. The severity of the applied stresses was evaluated based on an analysis of plant growth, plant color, and the content of photosynthetic pigments. Except for a strong wilting response caused by single salt and the combined treatment of cold and salt in *C. annuum* plants and a slight wilting response observed after all three treatments in *C. chinense* species, there were no visible signs of stress detectable at the end of the experiment (Fig. 1). However, image-based phenotyping of plants revealed stress-induced growth reduction and changes in plant color, expressed as excess greenness (ExG_plant_), in both species. Previous studies (Berger et al. 2012; Enders et al. 2019) also described the usability of plant imaging for growth analyses and the assessment of leaf color changes for estimation of stress tolerance levels in different genotypes. Using non-destructive plant imaging, we could show differences in stress-induced growth reduction over time, with *C. chinense* being more sensitive to the respective treatments than the cultivar of *C. annuum* (Fig. 2), which is in line with previous results (Genzel et al. 2021). In addition to that, analysis of the relative plant growth revealed that the two genotypes responded differently to the respective treatments. The combined stress treatment caused the strongest growth reduction, which was even significantly stronger compared to single cold in *C. chinense* (Fig. 2). An additive effect of the single stresses was in tendency also observed in *C. annuum* (Fig. 2).

Abiotic stress often causes accelerated senescence of older leaves or necrosis induced by increased osmotic stress (Berger et al. 2012) which results in changes in leaf color visible as yellowing (Signorelli et al. 2025). In this study, neither leaf senescence nor necrosis was visible. In accordance with that, the ExG_plant_, which represents the greenness of the plant obtained from image analysis, was not decreased by the treatments. On the contrary, the ExG_plant_ increased rapidly in *C. annuum* after single salt stress and even stronger after the combined treatment, whereas in *C. chinense* single cold stress and the combined treatment caused a similar response, which occurred earlier and stronger than for single salt treatment (Fig. 2) and stronger than in *C. annuum*. A loss of turgor, which was indicated by the wilting response (Fig. 1), and a reduction in leaf expansion, which affects the chlorophyll concentration, might explain the increased ExG_plant_ in both species (Rolando et al. 2015). It must be taken into account though that the ExG_plant_ reflects color changes of the whole plant; that color changes of individual leaves are not detected. Therefore, the leaf beneath the first branching was chosen for repeated analyses with a spectroradiometer throughout the experiment to monitor changes in leaf color via reflectance measurements and compare those to destructive pigment analyses on leaf level. Analysis of leaf reflectance represents a widely used sensitive non-destructive technique to monitor morphological and biochemical changes in plants or canopies (Penuelas and Filella 1998; Lowe et al. 2017) and for identification of abiotic and biotic stress responses (Herrmann et al. 2017; Camoglu et al. 2018). In fact, reflectance in the range between 500 and 590 nm, which includes the green spectrum (Lowe et al. 2017), showed the highest sensitivity to the stress treatments in both species (Fig. 3c, g). As for ExG_plant_, single cold and the combination of cold and salt increased the reflectance to a stronger extent than for single salt in both species and also to a greater extent in *C. chinense* than in *C. annuum* (Fig. 3a, c, e, g). A stress-induced increase of reflectance in the visible range, which is very sensitive to changes in foliar chlorophyll content (Gitelson and Merzlyak 1997), has also been described for cold-treated maize (Obeidat et al. 2018) and salt-treated castor bean (Li et al. 2010).

Many studies on non-destructive stress detection focus on plant responses to a single kind of stress (Behmann et al. 2014; Lara et al. 2016; Camoglu et al. 2018; Sytar et al. 2020). Here, we identified differences in response to single and combined stresses and also between the two species. Results of the pigment analysis indicate that salt treatment mitigated the strong reducing effect of the cold treatment on the chlorophyll and carotenoid content in both species, because the combined stress treatment reduced the chlorophyll content to a lower extent in both species (Tables 3, 4). This effect was even more pronounced in *C. annuum* (Table 3). Through the calculation of the first derivatives of reflectance (Fig. 3), with a specific focus on changes in the red edge region (690–750 nm), differences between the two species became more apparent. Single cold and the combination of cold and salt caused a clear shift of the red edge peak to shorter wavelengths in both species, with a stronger displacement of 12.4 nm observed in *C. chinense* compared to 5.2 nm (cold) and 3.5 nm (cold plus salt) in *C. annuum* (Fig. 3b, f, d, h) which corresponds to the significant chlorophyll loss in *C. chinense* (Tables 3, 4) and indicates a higher sensitivity to cold stress compared to *C. annuum* (Li et al. 2010; Lara et al. 2016). Furthermore, the correlation between chlorophyll content and values of the first derivative of reflectance was higher compared to the correlation between chlorophyll and original reflectance in both species (Fig. 5a, d). It has been shown that first derivative analysis facilitates the detection of critical wavelengths which makes it better suited for chlorophyll estimation than the analysis of the original reflectance data (Sonobe et al. 2020). Nevertheless, the correlation between leaf reflectance, first derivative as well as original reflectance, and chlorophyll content was much lower in *C. annuum* compared to *C. chinense*.

This observation might be linked to the different responses of the two species regarding the effect of single salt treatment on chlorophylls. While no effect of single salt treatment was seen in *C. chinense*, there was a significant salt-induced increase of chlorophylls detectable in *C. annuum* (Table 3). In general, decreased chlorophyll contents are observed in salt-sensitive genotypes and at high salt concentrations applied for a longer period of time (Hand et al. 2017). Although salinity often results in a reduction of chlorophyll concentration (Siddiqui et al. 2020; Cha et al. 2021), increased foliar concentrations of chlorophyll under saline conditions have been observed by different authors as well (Lacerda et al. 2006; Li et al. 2010). The reaction of the chlorophyll content to salinity is also dynamic. In accordance, low salt concentrations induced an increase, whereas higher salt concentrations reduced the content of chlorophylls in *Vicia faba* (Hamada and El-Enany 1994). Different hypotheses for an increased chlorophyll content under saline conditions have been proposed. Li et al. (2010) suggested a growth-inducing effect of low salt concentrations based on an increased chlorophyll content; however, the decreased RGR_PLA_ observed after single salt treatment in this study contradicts this assumption. Lacerda et al. (2006) assign the increased chlorophyll content to a number of morpho-physiological responses of the plant to alleviate negative effects caused by salt stress. Also, it has been suggested that an increased concentration of chlorophyll under saline conditions is a response to the osmotic stress caused by the salt, which can be explained by an altered chloroplast development and possibly increased thylakoid number per chloroplasts (García-Valenzuela et al. 2005). In fact, *C. annuum* treated with single salt strongly wilted, indicating that the plants experienced osmotic stress. Despite this relatively pronounced wilting response, *C. annuum* showed a delayed significant reduction of RGR_PLA_ (Fig. 2a, b) and seems to be more tolerant to salt stress than *C. chinense*.

Furthermore, the content of carotenoids was significantly increased in the leaves of *C. annuum* after 7 days of salt treatment (Table 3), while there was no significant change detectable in *C. chinense* (Table 4). Carotenoids protect the plant's photosynthetic apparatus under abiotic stress acting as scavengers of reactive oxygen species and via dissipation of excessive excitation energy (Maoka 2020). Changes in the ratio of carotenoids to chlorophylls can serve as an indicator of altered photosynthetic activity, development, and response to environmental stresses (Gitelson 2020). The significant increase of the carotenoid to chlorophyll ratio, an indicator of plant stress or senescence (Penuelas and Filella 1998), was only observed in *C. annuum* plants treated with single cold and in *C. chinense* plants treated with the combination of cold and salt after 14 days. Moreover, the chlorophyll a to chlorophyll b ratio, which can be altered in changing environmental conditions (Sonobe et al. 2020), was only significantly increased in *C. annuum* after 14 days of single cold treatment. An increase of the chlorophyll a to chlorophyll b ratio has been observed in response to chilling before (Yang et al. 2009). The increased chlorophyll a to chlorophyll b ratio might be linked to stress tolerance. It was suggested that the transformation of chlorophyll b to chlorophyll a enables the plant to maintain a relatively high chlorophyll a content allowing an adaptation to low temperatures (Yang et al. 2009). However, in this study the contents of chlorophyll a and b decreased significantly due to cold treatment in both species (Table S2) indicating that both species are sensitive to low temperatures. In contrast to *C. annuum*, *C. chinense* had also significantly lower contents of chlorophyll a and b after the combined treatment of cold and salt. Taking RGR_PLA_ and ExG_plant_ into account as well, *C. chinense* shows a higher sensitivity to the cold treatment, single and in combination with salt, than *C. annuum*, which is in accordance with our previous study (Genzel et al. 2021). A higher sensitivity of *C. chinense* to the applied stresses is in line with the aforementioned stronger shift of the red edge peak of the first derivative to the left (Fig. 3f, h). The separation between tolerant and sensitive genotypes via analysis of leaf reflectance has been described for cotton lines of different sensitivity to waterlogging via analysis of leaf reflectance in the range of 550–700 nm (Pan et al. 2019). Salt-induced changes of leaf reflectance in salt-sensitive genotypes of wheat were much stronger compared to changes in salt-tolerant genotypes as well (Moghimi et al. 2018).

Abiotic stress tolerance is also linked to the content of protective compounds like phenolic compounds (Sharma et al. 2019), which tend to increase in *Capsicum* under salt and cold stress conditions (Hand et al. 2017; Genzel et al. 2021). In accordance with a higher stress tolerance level assumed for the *C. annuum*, the contents of total phenolics and flavonoids were higher compared to the *C. chinense* species (Table 3). In both species, stress treatments induced an accumulation of phenolic compounds with the combination of stresses showing the strongest impact, followed by single cold and single salt (Tables 3, 4). The non-destructive assessment of flavonoids based on hyperspectral reflectance has gained rather low attention compared to the determination of chlorophylls and carotenoids. Based on strong absorption peaks within the UV and in the blue region, indices including wavelengths within these regions have been proposed for the determination of flavonoids (Sytar et al. 2020). In previous studies, the index FLAV_700,760_ correlated well with the flavonoids content and with the content of chlorogenic acid (Quemada et al. 2014; Sytar et al. 2020). In *C. chinense*, changes of the FLAV_700,760_ of stressed plants relative to control plants corresponded to the detected accumulation of total flavonoids (Fig. 4); however, correlations between TP or TF and FLAV_700,760_ were not significant. Although significant correlations between phenolic compounds and FLAV_700,760_ were detected in *C. annuum* (Fig. 6), these correlations were not as high as described in previous studies on the assessment of phenolic compounds via spectral leaf reflectance (Cotrozzi and Couture 2020; Sytar et al. 2020). It has to be considered that phenolic compounds represent a group of different molecules of different structures and that the two *Capsicum* species also differ regarding the composition of these compounds (Genzel et al. 2021), which might affect the correlation between spectral reflectance indices and contents of total phenolics and flavonoids. In line with this, it has been shown that the FLAV_700,760_ is suitable for determining phenolic acids like chlorogenic acid for instance, whereas contents of flavonol glycosides like kaempferol-3-glucuronide did not correlate with this index (Sytar et al. 2020). Moreover, the present study investigated leaf reflectance in the wavelength range between 325.8 and 789.9 nm. However, higher wavelength ranges might have been better suited, because good correlations between the content of phenolic compounds and absorption features were also described within the range of 1100–2400 nm (Cotrozzi and Couture 2020). The FLAV_700,760_ showed also a significant correlation with chlorophylls and carotenoids in *C. chinense* (Fig. 6) which is in line with the described suitability of red edge reflectance indices for chlorophyll estimation (Zarco-Tejada et al. 2001).

Most of the vegetation or stress indices from datasets of hyperspectral measurements are commonly used to estimate changes in chlorophyll concentration in order to study the impact of stress on plants (Gitelson and Merzlyak 1997; Penuelas and Filella 1998; Zarco-Tejada et al. 2001; Lowe et al. 2017). In the present study, selected indices were investigated regarding their applicability to detect changes in the concentration of photosynthetic pigments, but also of total phenolics and total flavonoids upon stress treatment of *Capsicum* species. In *C. chinense* plants, applied stress treatments significantly affected all tested indices, whereas only RGI, NDRE, NDVI_green_, and ZMI were significantly affected in *C. annuum* (Fig. 4). Again, single cold and the combined treatment had a stronger effect than single salt. In previous studies, the indices NDRE (Barnes et al. 2000), NDVI (Gamon and Surfus 1999), NDVI_green_ (Poss et al. 2006), ZMI (Zarco-Tejada et al. 2001), and GM1 (Gitelson and Merzlyak 1997) were strongly correlated with the chlorophyll content in leaves. In this study however, the correlation with photosynthetic pigments was much lower in *C. annuum* than in *C chinense*, with only PRI showing a significant correlation with the content of chlorophylls and carotenoids (Fig. 6). This might be explained by the higher content of phenolic compounds in *C. annuum* compared to *C. chinense*, since it has been shown that high contents of flavonoids reduce the accuracy of chlorophyll estimation (Falcioni et al. 2020). In addition to that, when the correlation was analyzed separately for each stress treatment, it became obvious that a single type of stress affected the correlation differentially (Fig. S3). In *C. chinense*, many of the investigated spectral indices significantly correlated with the content of chlorophylls (Fig. S3) only if single cold or its combination with salt were analyzed. In *C. annuum*, however, there was no correlation observed between the content of chlorophylls and spectral indices, except for PRI when only single cold treatment was regarded. This shows that the specific stress treatment as well as the species affects the suitability of spectral indices for the assessment of plant stress responses. Wang et al. (2025) also demonstrated that the correlation between spectral features and photosynthetic stress parameters is different for different stresses and their combinations. Partly it seemed as if the combination of both stresses affected or even masked the effect of the single treatments as it was e.g. observed in *C. chinense* for the content of chlorophylls, which was decreased to a lesser extent by the combined stress compared to single cold stress. Altogether, correlation analyses resulted in very different results regarding the suitability of spectral indices for the estimation of the analyzed physiological stress parameters. Still, using the index PRI, significant correlations with the content of chlorophylls and the ExG_plant_ were obtained for both species. In addition, PRI was the only index which allowed separation between all three stress treatments in both species (Fig. 4). Thus, the accumulating effect of the single stresses could be nicely seen in *C. chinense*. Furthermore, the temporal changes of the index PRI upon the different treatments (Fig. 7) corresponded to the changes of the photosynthetic pigments.

Combined abiotic stress reactions are categorized into positive and negative interactions, either mitigating or increasing damage or lethality of the stresses in combination. In this study, the impact of the combination of both stresses in comparison to the single stress application is rather low and dependent on the *Capsicum* species. In *C. chinense*, the effect of single cold stress is dominating the observed effects for e.g. chlorophyll content, leaf color, and hyperspectral reflectance changes. In *C. annuum*, however, there is also a strong effect of single salt stress detectable, which is sometimes similar to the combined treatment as e.g. relative growth and plant greenness. An additive effect of single stresses was detected for *C. chinense,* where both single stresses affected the growth to a similar extent and resulted in a stronger reduction when applied in combination. Also, the accumulation of phenolics and flavonoids was further increased by the combination of both treatments in both species. This induction might prevent the degradation of photosynthetic pigments as these aromatic secondary metabolites might protect the plant cell from reactive oxygen species (ROS) (Jan et al. 2022). For the changes of the photosynthetic pigments, an additive effect of both single stresses was also observed, but due to the contrasting effects of single cold and single salt on chlorophylls and carotenoids, the strong reducing effect of single cold was diminished when applied in combination with salt. Although single salt increased the content of photosynthetic pigments only in *C. annuum*, the parallel application of salt stress might have mitigated the loss of photosynthetic pigments seen in single cold stress leading to a lower reduction under combined stress for both species. A weakened negative effect on the photosynthetic apparatus under combined salt and cold treatment compared to single stress has also been described for faba bean (El-Dakak et al. 2023). In contrast to this study, the authors described mitigation of the negative effect caused by salt due to cold stress.

Although salt stress has been described as a stress factor in different stress combination scenarios (Mittler 2006), the interaction of salt and cold stress has not been investigated much so far. The study of the combination of salt and cold stress is important, because a parallel occurrence of soil salinity or saline irrigation water and temperature stresses is very likely under usual growing conditions. A prediction of the impact of the combination of stresses cannot be simply deduced by superimposition of known mechanisms of the single stress responses (Jiang et al. 2025); however, some differences and similarities between the combined stresses might support the observations seen. Cold stress as well as salt stress causes reduced water availability (Serrano et al. 2017; Soualiou et al. 2022) which is shown in the wilting response of the two species. Also, most stresses cause an induction of ROS, which can be further increased under stress combinations (Genzel et al. 2021; Zandalinas et al. 2021). Consistently, Zandalinas et al. (2021) identified ROS homeostasis as one of the mechanisms involved in the common response in various stress combinations. The stronger induction of flavonoid content observed under combined stress in this study would indicate a stronger ROS accumulation, inducing increased flavonoid biosynthesis. However, many mechanisms of combined stress responses are also different from those to single stresses as seen for unique genes expressed only in response to combined stresses, but not in response to the single stresses (Zandalinas et al. 2021).

Hyperspectral assessment of plant responses to stress combinations is rather challenging and the accuracy for prediction of plant stress parameters via spectral data decreases with increasing stress combinations and environmental variation (Cotrozzi and Couture 2020). Within the last years, a few studies investigating the applicability of hyperspectral assessments for combination or multi-stresses have been published (Ma et al. 2018; Cotrozzi and Couture 2020; Ejaz et al. 2023; Wang et al. 2025), however, to our knowledge the present study is the first one analyzing the combined effect of salt and cold.

## Conclusion

In this study, image-based phenotyping and hyperspectral leaf reflectance detected cold, salt, and combined stress in *Capsicum* plants before stress symptoms were clearly visible. The combination of salt and mild cold stress reduced the growth of *C. chinense* to a greater extent than single cold stress; however, there were no further negative additive stress effects detected in both investigated species. In contrast, the impact of single stress treatment, mostly single cold stress, was often rather comparable to the combined stress treatment. Although cold-induced chlorophyll loss corresponded well to the increased leaf reflectance around 500 nm and to the shift of the red edge peak to lower wavelengths, a clear distinction between single cold and the combined stress treatment was not given by the hyperspectral data for either of the species. However, changes in the leaf spectral signature were indicative of the severity of stress, since the level of displacement of the peak seemed to correlate with stress severity allowing a separation between the less tolerant *C. chinense* species and the more tolerant *C. annuum*. Among the studied vegetation indices, the PRI was the most promising index for detection of differences in the response of the two species to single and combined stress treatments. Our results showed that the use of hyperspectral signals for quantification of the responses to different stresses must be done with caution as the changes of the spectra overlap and might reduce the final output of the signal depending on the impact of single stresses. Additional processing and analysis of hyperspectral data using e.g. partial least-squares discriminate analysis might be suitable to address this problem and improve differentiation between stress treatments (Cotrozzi and Couture 2020; Wang et al. 2025). All in all, further research on combined stresses is essential to propel research into the breeding of climate-resilient crops and to contribute to future food security.

## Supplementary Information

Below is the link to the electronic supplementary material.

Supplementary file1 (DOCX 906 KB)

## Data Availability

The experimental data that support the findings of this study are available on PLANTdataHUB (Weil et al. 2023) (https://git.nfdi4plants.org/usadellab/Cold_salt_stress_Capsicum).

## Abbreviations

ExG_plant_: Excess greenness of the plant
NDRE: Normalized difference red edge index
NDVI: Normalized difference vegetation index
PLA: Projected leaf area
PRI: Photochemical reflectance index
RGR_PLA_: Relative growth rate
SLA: Specific leaf area
TF: Total flavonoids
TP: Total phenolics
