# Supplementary material for: Comparison of single and combined salt and cold stress effects and their challenges for hyperspectral measurements of different Capsicum species
Source: Planta. 2025 Nov 8;262(6):147. doi: 10.1007/s00425-025-04865-0 (PMC12598683; doi:10.1007/s00425-025-04865-0)
Supplement: Supplementary file 1 — Supplementary file1 (DOCX 906 KB) [file 425_2025_4865_MOESM1_ESM.docx]

**Supplementary Information**

**Comparison of single and combined salt and cold stress effects and their challenges for hyperspectral measurements of different *Capsicum* species**

Franziska Genzel^1,2^, Anika Wiese-Klinkenberg^1,2^

^1^Institute of Bio- and Geosciences, Bioinformatics (IBG-4) and Plant Sciences (IBG-2), Forschungszentrum Jülich GmbH, 52425 Jülich, Germany,

^2^Bioeconomy Science Center (BioSC), Forschungszentrum Jülich GmbH, 52425 Jülich, Germany

**Table S1** Management of temperature and light regime in growth chamber

| Parameter | Time | Set | | | |
| --- | --- | --- | --- | --- | --- |
|  |  | Control | Cold | Salt | Cold+Salt |
| Temperature | 00:00 | 18 | 12 | 18 | 12 |
|  | 05:30 | 18 | 12 | 18 | 12 |
|  | 06:00 | 24 | 18 | 24 | 18 |
|  | 16:00 | 24 | 18 | 24 | 18 |
|  | 16:30 | 18 | 12 | 18 | 12 |
|  | 23:59 | 18 | 12 | 18 | 12 |
| Lamps | 00:00 | off | off | off | off |
|  | 06:00 | on | on | on | on |
|  | 16:01 | off | off | off | off |
|  | 23:59 | off | off | off | off |


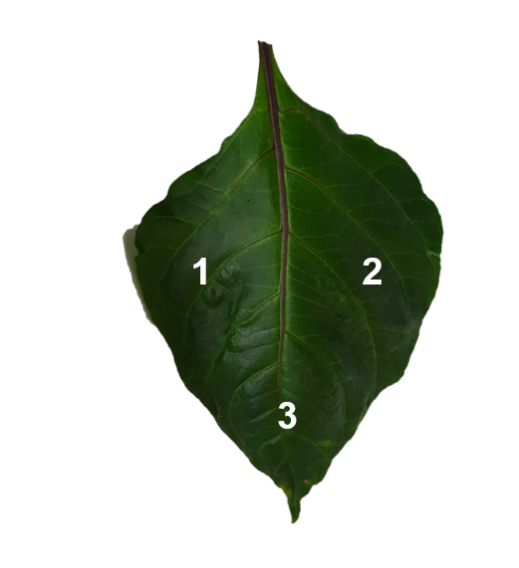


**Fig. S1** Positions used for spectral reflectance measurement with the spectroradiometer.

**
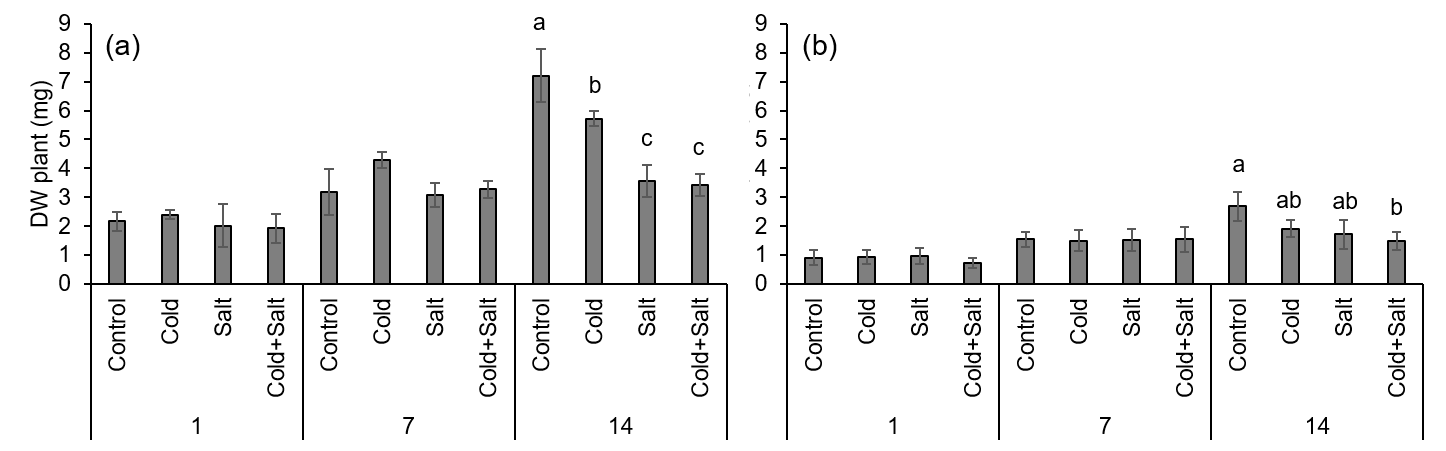
Fig. S2** Dry weight (DW, mg) of *C. annuum* (**a**) and *C. chinense* plants (**b**) shown for control, cold, salt, and cold plus salt 1, 7, and 14 days after start of stress treatment. Data show means ± standard deviations (*n* = 4 plants). Different letters indicate significant differences between treatments (control, cold, salt, cold plus salt) per species within each time point (*P* ≤ 0.05)

**Table S2** Foliar concentration of chlorophyll a and chlorophyll b of *C. annuum* and *C. chinense* grown under different conditions (control, cold, salt, cold plus salt) after 1, 7, and 14 days. Data show means ± standard deviations (*n* = 4 plants). Significant differences between treatments within each time point are indicated by different letters (*P* ≤ 0.05)

| Pigment concentration (mg/g DW) | Day | Control | Cold | Salt | Cold+Salt |
| --- | --- | --- | --- | --- | --- |
| *C. annuum* | | | | | |
| Chlorophyll a | 1 | 14.0 ± 0.8 a | 12.5 ± 0.8 ab | 16.7 ± 1.6 b | 13.8 ± 1.7 ab |
|  | 7 | 13.0 ± 0.3 a | **7.9 ± 0.6 b** | **16.2 ± 1.8 c** | 11.4 ± 0.9 a |
|  | 14 | 11.5 ± 0.8 ac | **6.7 ± 0.4 b** | **14.4 ± 1.4 c** | 9.9 ± 2.2 ab |
| Chlorophyll b | 1 | 2.2 ± 0.2 | 1.8 ± 0.2 | 2.6 ± 0.5 | 2.1 ± 0.4 |
|  | 7 | 1.6 ± 0.05 ac | **1.0 ± 0.2 b** | 2.2 ± 0.4 a | 1.4 ± 0.1 bc |
|  | 14 | 1.9 ± 0.1 ac | **0.8 ± 0.04 b** | 2.2 ± 0.4 c | 1.4 ± 0.4 ab |
| *C. chinense* | | | | | |
| Chlorophyll a | 1 | 9.4 ± 1.00 | 8.7 ± 1.0 | 9.4 ± 1.2 | 8.4 ± 1.0 |
|  | 7 | 9.7 ± 0.8 a | **6.1 ± 1.0 b** | 9.4 ± 1.5 a | 7.5 ± 0.7 ab |
|  | 14 | 9.5 ± 0.6 a | **5.2 ± 0.4 b** | 8.5 ± 1.6 ac | **6.4 ± 0.4 bc** |
| Chlorophyll b | 1 | 1.2 ± 0.1 | 1.1 ± 0.2 | 1.2 ± 0.2 | 1.0 ± 0.1 |
|  | 7 | 1.2 ± 0.1 a | **0.6 ± 0.1 b** | 1.2 ± 0.3 a | 0.9 ± 0.2 ab |
|  | 14 | 1.4 ± 0.1 a | **0.6 ± 0.1 b** | 1.2 ± 0.2 a | **0.8 ± 0.1 b** |


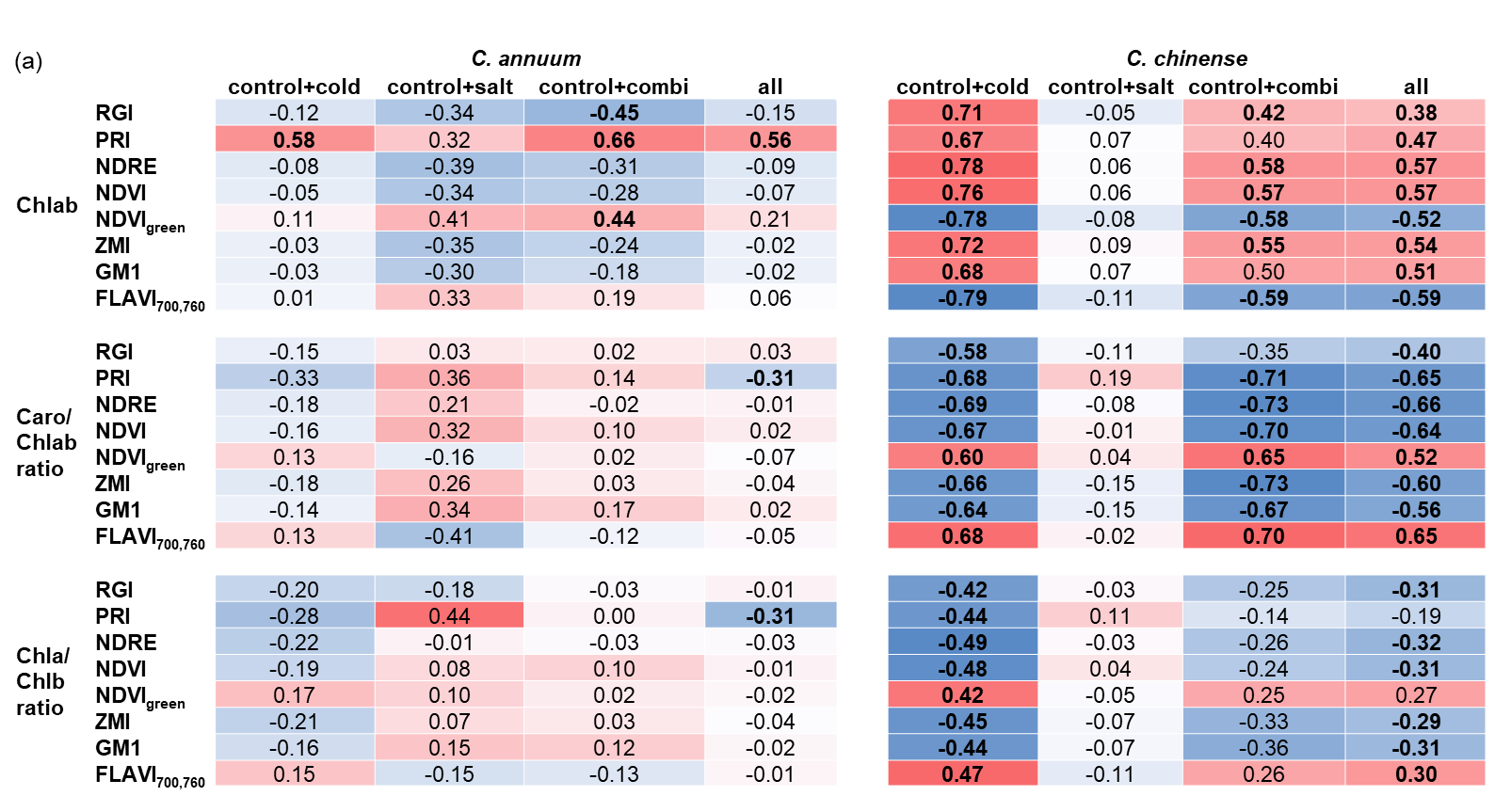

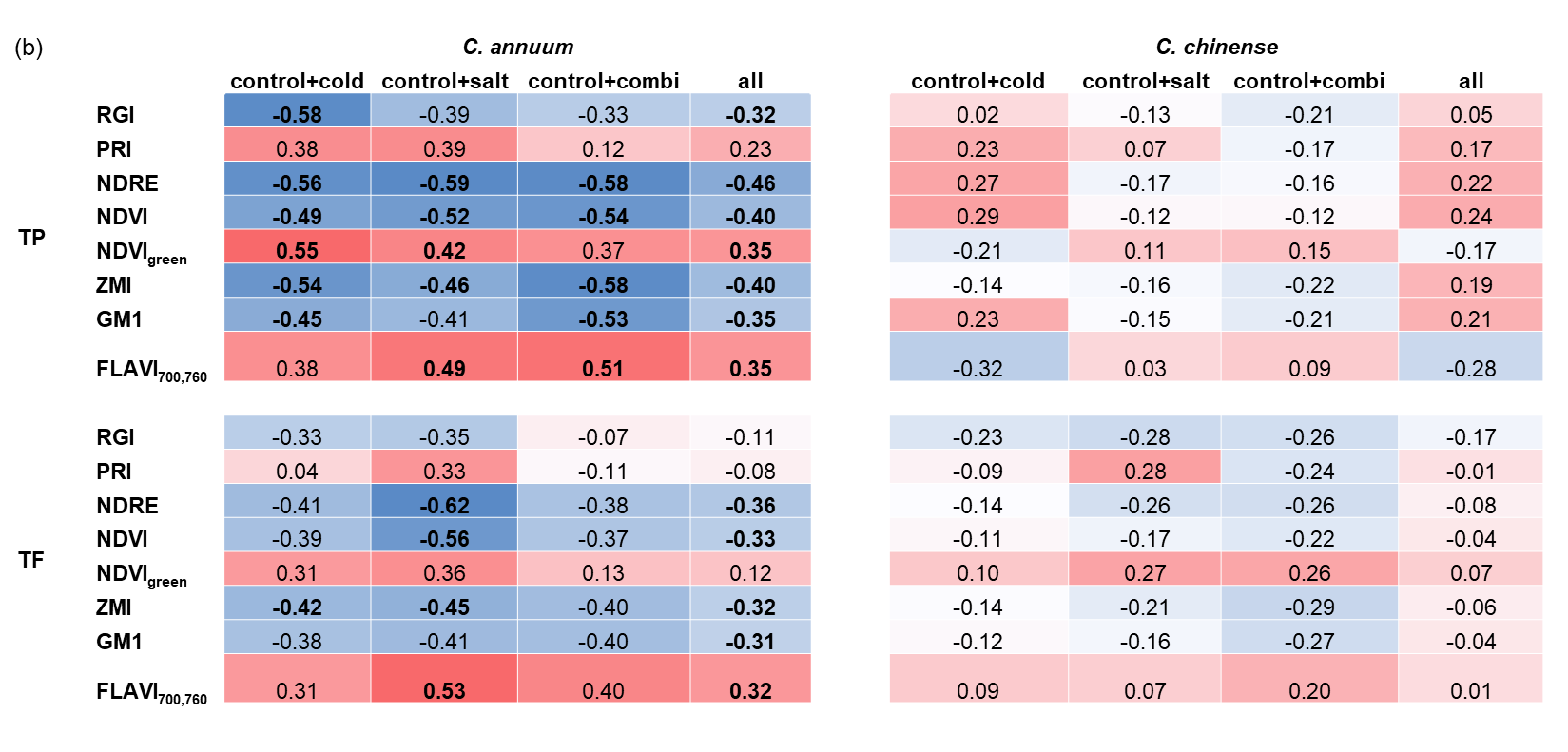


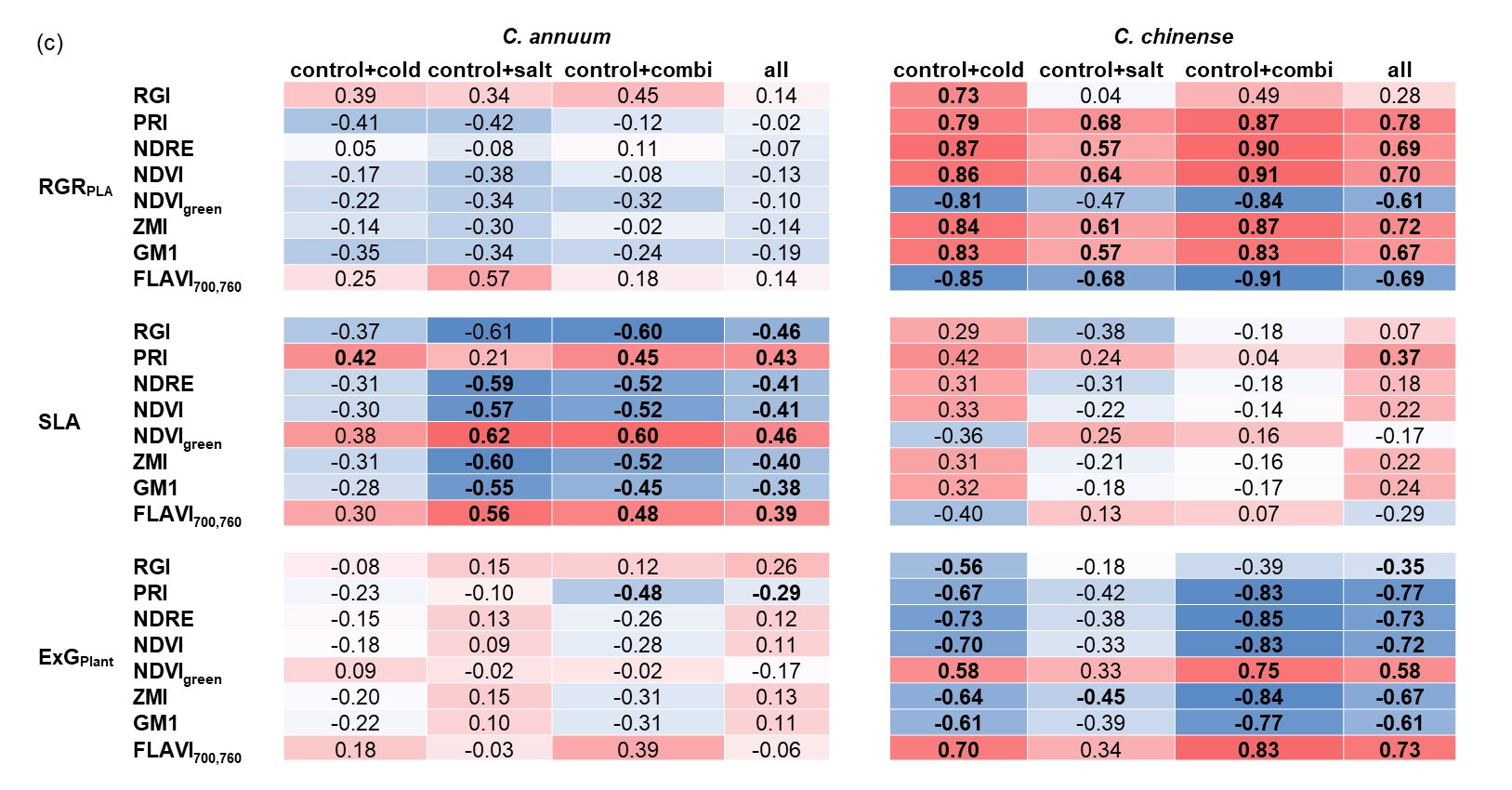


**Fig. S3** Results of Pearson correlation conducted separately for the respective treatments (control and cold, control and salt, control and combination, all together). Shown is the coefficient (R, Pearson) of determination of relationships between reflectance indices and stress indicators [(**a**) chlorophyll ab (Chlab, mg/g DW), carotenoids (Caro, mg/g DW), the ratio of carotenoids to chlorophyll ab (Caro/Chl ratio), the ratio of chlorophyll a to b (Chl a/b ratio), (**b**) total phenolics (TP, mg/g DW), total flavonoids (TF, mg/g DW), (**c**) relative growth rate (RGR_PLA_, %/day), specific leaf area (SLA, cm^2^/mg), and Excess Greenness Index (ExG_plant_)] of *C. annuum* and *C. chinense* including data from 1,7,and 14 days after start of stress treatment. Significance (*P* ≤ 0.05) is indicated in bold
